# Virtual Screening and Biological Evaluation of Piperazine Derivatives as Human Acetylcholinesterase Inhibitors

**DOI:** 10.1155/2013/653962

**Published:** 2013-10-28

**Authors:** Kavitha Raj Varadaraju, Jajur Ramanna Kumar, Lingappa Mallesha, Archana Muruli, Kikkeri Narasimha Shetty Mohana, Chethan Kumar Mukunda, Umesha Sharanaiah

**Affiliations:** ^1^Postgraduate Department of Biochemistry, JSS College, Ooty Road, Mysore 570025, Karnataka, India; ^2^Postgraduate Department of Chemistry, JSS College, Ooty Road, Mysore, India; ^3^Department of Chemistry, University of Mysore, Manasagangothri, Mysore, India; ^4^Department of Biotechnology, University of Mysore, Manasagangothri, Mysore, India

## Abstract

The piperazine derivatives have been shown to inhibit human acetylcholinesterase. Virtual screening by molecular docking of piperazine derivatives 1-(1,4-benzodioxane-2-carbonyl) piperazine (K), 4-(4-methyl)-benzenesulfonyl-1-(1,4-benzodioxane-2-carbonyl) piperazine (S1), and 4-(4-chloro)-benzenesulfonyl-1-(1,4-benzodioxane-2-carbonyl) piperazine (S3) has been shown to bind at peripheral anionic site and catalytic sites, whereas 4-benzenesulfonyl-1-(1,4-benzodioxane-2-carbonyl) piperazine (S4) and 4-(2,5-dichloro)-benzenesulfonyl-1-(1,4-benzodioxane-2-carbonyl) piperazine (S7) do not bind either to peripheral anionic site or catalytic site with hydrogen bond. All the derivatives have differed in number of H-bonds and hydrophobic interactions. The peripheral anionic site interacting molecules have proven to be potential therapeutics in inhibiting amyloid peptides aggregation in Alzheimer's disease. All the piperazine derivatives follow Lipinski's rule of five. Among all the derivatives 1-(1,4-benzodioxane-2-carbonyl) piperazine (K) was found to have the lowest TPSA value.

## 1. Introduction

Acetylcholinesterase (AChE) hydrolyses acetylcholine is associated with nerves and muscles mainly found in synapses. AChE plays an important role in regulation of cholinergic function. It has been shown to be involved in dysfunction of the central cholinergic system in Alzheimer's disease (AD). It is a progressive neurodegenerative disorder, characterized by an impairment of cognitive function leading to dementia. The main characteristic features of the disease include *β*-amyloid (A*β*) plaques, neurofibrillary tangles, and their by synaptic loss. AD is estimated to account for about 50–60% dementia cases, in persons over 65 years of age [1]. Symptoms of Alzheimer's disease include memory loss, language deficit, depression, agitation, and mood disturbances [2–4]. But the exact cause for AD is still not known. Several hypotheses tried to explain the cause of the disease [5]. Among those, the oldest, on which most currently available drug therapies are based, is the cholinergic hypothesis, which proposes that AD is caused by reduced synthesis of the neurotransmitter acetylcholine [1]. Even though the hypothesis failed to get widespread support, but it can be stated that cholinergic scarcity is responsible for the symptoms of AD [5]. This led to the designing and synthesis of AChE inhibitors. The inhibition causes an increase in the concentration of acetylcholine in cholinergic synapse. This might ameliorate the disease symptoms of AD [6, 7]. Tacrine, Donepezil, Rivastigmine, and Galanthamine are so far approved drugs by Food and Drug Administration (FDA) to treat AD in the US. Alzheimer's disease (AD) accounts for 50% of the cases of dementia in elderly people and there are currently 2.5 to 4.0 million estimated Alzheimer's disease patients in the United States and some 17 to 25 million worldwide [8, 9].

Piperazine is currently the most important building blocks in drug discovery with a high number of positive hits encountered in biological screens of this heterocycle and its congeners. A literature survey revealed that piperazine derivatives are important pharmacophores across a number of different therapeutic areas [10] and they act as antifungal [11], antipsychotic [12], antimicrobial [12, 13], antioxidant [13], antimalarial, and anti-HIV protease [14]. 1,4-Benzodioxane-2-carboxylic acid (BCA) is a very important entity in medicinal chemistry since it was chiral building blocks in the design and synthesis of chiral therapeutic agents [15]. 

Highly efficient resolutions of BCA with para substituted 1-phenylethylamines [16], crystallographic, theoretical, and morphologic approach of (S)-1-phenylethylamine para substitution on the resolution of 1,4-benzodioxane-2-carboxylic acid have been reported [17]. Recently, BCA was found to be a potent, selective, and orally active prostaglandin D2 (PGD2) 4 receptor antagonist [15]. Enzymatic resolution of ethyl 1,4-benzodioxane-2-carboxylate catalyzed by a microbial esterase has been reported to produce optically active BCA in good yield [18]. 

Presently, many drugs are available in the market like Rivastigmine (Exelon), Donepezil (Aricept), Galantamine (Reminyl), and Tacrine (Cognex). However, these drugs have been reported for adverse side effects like vomiting, diarrhea, hives, and liver toxicityni respectively. Several investigators have synthesized AChE inhibitors like alkylpyridium polymers [19], dehydroevodiamine (DHED) [20], N-aryl-substituted succinimides [21], lycorine derivatives [22], salicylanilide N-alkylcarbamates [23], and 7-methoxytacrine-adamantylamine heterodimers [24].

It is well known that AChE possesses two binding sites for the neurotransmitter acetylcholine. Specifically, the active center site that is located at the bottom of a 20 Å gorge, and the peripheral binding site that is rich in hydrophobic residues and is located at the rim of the gorge on the surface of the enzyme [25, 26].

Accumulated evidence suggests that the present prescribed drugs for the symptomatic treatment of AD have a tendency of binding to both the peripheral anionic site (PAS) and the catalytic site of AChE. Among these sites, the PAS site is gaining more interest in designing novel drugs as the site is involved in catalytic site allosteric modulation and also has importance in noncholinergic functions like cell adhesion, neurite outgrowth in developing and transformed neural cells [28–31] and amyloidosis through an interaction with the amyloid *β*-peptide in AD [32, 33], and it also has been shown to interact with an omega loop on an adjacent AChE subunit [34]. 

PAS site clustered around the entrance of the catalytic site gorge. It is also associated with a number of surface loops and enzymatically involved residues. Propidium or fasciculin, decidium, ethidium, gallamine, and drugs like donepezil, galantamine majorly bind to this site [30, 32, 35–40]. Based on present research interest, the hybrids of known marketing drugs also have reported as potent AChE inhibitors and they too have a binding affinity towards the PAS site [41]. But because of bioavailability problems and possible side effects, there is still great interest in finding better AChE inhibitors. 

In the present paper we have reported AChE inhibition and virtual screening for chemically synthesized novel piperazine derivatives to both PAS site and catalytic site of huAChE and also studied the mechanism of interactions by computational analysis. Furthermore, these derivatives were also analyzed for drug likeness and permeability through intestine and blood brain barrier. 

## 2. Experimental

### 2.1. Materials and Methods

All solvents and reagents were purchased from Sigma and Merck chemicals. All the piperazine compounds were synthesized according to the literature procedure [13]. 

#### 2.1.1. AChE Inhibition Assay

The inhibitory effects of the compounds Piperazine-K, Piperazine-S1, Piperazine-S3, Piperazine-S4, and Piperazine-S7 obtained here were tested on AChE in vitro by using Ellman's method [42]. 

The Ellman's reaction for assay of AChE involves the use of a thiol reagent, namely, 5,5′-dithiobis(2-nitrobenzoic acid) (DTNB), also known as Ellman's reagent, which is reduced by the thiocholine generated by enzymic hydrolysis of acetylthiocholine (ATCh) to yield the chromophore 2-nitro-5-thiobenzoic acid. The assay solution consisted of 0.1 M PBS buffer (pH 8.0) (3.0 mL), with the addition of 0.01 M 5,5′-dithiobis(2-nitrobenzoic acid) (100 *μ*L) (DTNB, Ellman's reagent), 10 *μ*L substrate (Acetylthiocholine iodide, 0.075 M), enzyme (human erythrocyte acetylcholinesterase 1 *μ*g in 0.1 M PBS buffer of pH 8.0) (50 *μ*L), and different concentrations of derivatives. Incubate for 6 minutes, at room temperature with continuous gentle shake. Wait until the yellow color develops and measure at 412 nm.

#### 2.1.2. Ligand Preparation for Docking

The structures were drawn by Chemsketch (http://www.acdlabs.com/resources/freeware/chemsketch/) and converted to protein data bank (PDB) file format by using Openbabel software (http://www.openbabel.org/). Ligand preparation includes the addition of hydrogen atoms, neutralization of the charge groups, and removal of any miscellaneous structures from the ligand. Prepared and optimized structures of ligands were used for molecular docking. 

#### 2.1.3. Selection and Preparation of Receptor Protein

For molecular modeling, Blastp was done for (GenBank: AAA68151.1) acetylcholinesterase (Homo sapiens) with 614 amino acids. 100% similarity was found with 4EY4 PDB file. This PDB structure of acetylcholinesterase at 2.16 Å resolutions was retrieved from the Research Collaboratory for Structural Bioinformatics (RCSB) Protein Data Bank (PDB) (http://www.rcsb.org/). Then, water molecules, metal ions, cofactors, and ligands were removed and used for docking. 

#### 2.1.4. Binding Site Prediction

Binding sites were characterized by CASTp, ligsite, and Q-Site finder and compared by an extensive literature search. By comparing prediction of CASTp algorithm, ligsite, and Q-Site Finder, best binding sites were selected. CASTp method was used to identify and measure the binding sites, active sites, surface structural pockets (accessible), interior cavities (inaccessible), shape (alpha complex and triangulation), area and volume (solvent and molecular accessible surface) of each pockets, and cavities of proteins. CASTp could be used to measure the number, area, circumference of mouth openings of each pocket in solvent, and molecular accessible surface. Ligsite is a new program for the automatic and time-efficient detection of pockets on the surface of proteins that may act as binding sites for small molecule ligands and also is able to identify the binding sites of small molecule ligands with high precision. Q-site finder was used to predict the ligand binding site on a protein. It involves the binding of hydrophobic probes to proteins, searching probe clusters on the protein with favorable binding energy, and arranging them in an order according to the binding energy of each cluster.

#### 2.1.5. Molecular Docking

The docking of acetylcholinesterase was performed against piperazine derivatives by using Autodock 4.2 (http://autodock.scripps.edu/wiki/AutoDock4/), a novel and robust automated docking method. Possible favorable interactions (of minimum binding energy and hydrogen bonding) with amino acids at possible target sites (PAS and catalytic site) were determined by Lamarckian genetic algorithm (LGA, method which is the most efficient, reliable, and successful) and Genetic Algorithm. Autodock 4, combines energy evaluation through grids of affinity potential employing various search algorithms to find the suitable binding position for a ligand on a given protein [43].

Prepared ligands were docked within the grid region (grid size was set to 60∗60∗60 points with grid spacing of 0.375 Å) which was set to the centre of active gorge of AChE and also to the PAS site separately. Ten independent docking runs were carried out for each ligand and results were clustered according to the 1.0 RMSD criteria. The lowest energy cluster returned by Autodock for each derivative was used for further analysis. All other parameters were maintained at their default settings. 

#### 2.1.6. Calculation of Molecular Properties

The molecular properties were calculated on the basis of simple molecular descriptors used by “Lipinski's rule of five” [44, 45]. The five properties consist of molecular weight, hydrogen bond donor, hydrogen bond acceptor, log *P*, and number of rotatable bonds. The other significant property called total polar surface area (TPSA) [46] metric for the optimization of a drug's ability to penetrate through intestinal and blood brain barrier was also calculated using the online chemoinformatics software molinspiration (http://www.molinspiration.com/).

## 3. Results and Discussion

Acetylcholine is the most abundant primary neurotransmitter in brain, responsible for cholinergic function. AChE plays important role in hydrolysis of acetylcholine. To date, the available drug treatment for AD has been based on the reduction of cognitive impairment by enhancing cholinergic neurotransmission by acetylcholine and in turn inhibiting AChE activity. 

**Figure 1:**
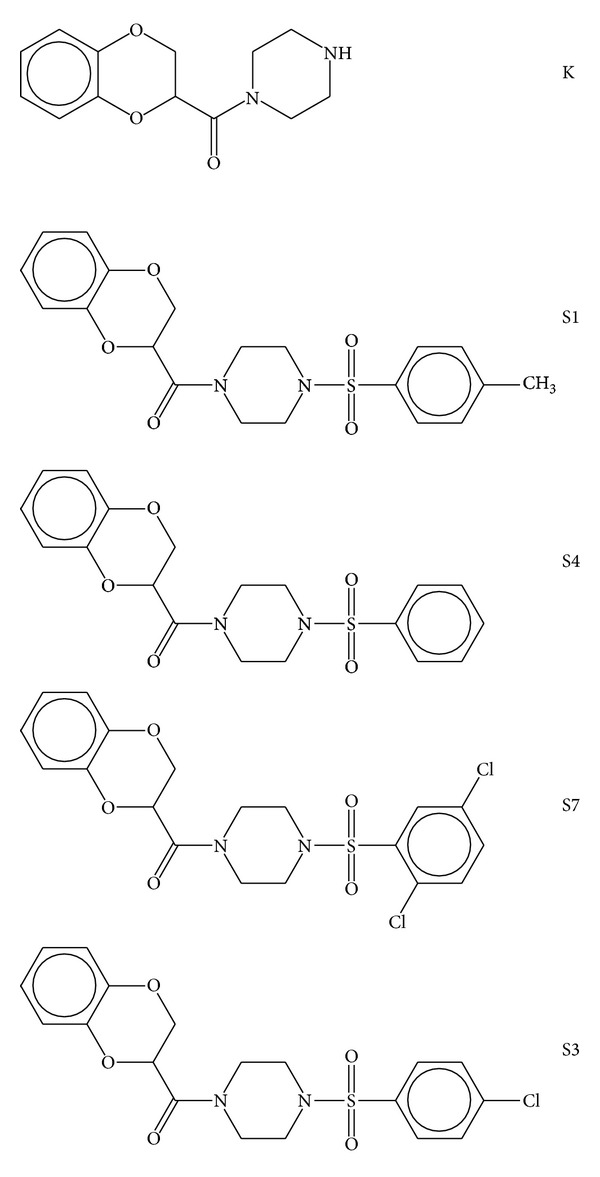
Ligand structures: 1-(1,4-benzodioxane-2-carbonyl) piperazine (K), 4-(4-methyl)-benzenesulfonyl-1-(1,4-benzodioxane-2-carbonyl) piperazine (S1), 4-benzenesulfonyl-1-(1,4-benzodioxane-2-carbonyl) piperazine (S4), 4-(2,5-dichloro)-benzenesulfonyl-1-(1,4-benzodioxane-2-carbonyl) piperazine (S7), and 4-(4-chloro)-benzenesulfonyl-1-(1,4-benzodioxane-2-carbonyl) piperazine (S3).

1-(1,4-Benzodioxane-2-carbonyl) piperazine (K) was synthesized from 1,4-benzo dioxan-2-carbonyl chloride with piperazine in dry DMF at 80°C for 8 h. The progress of the reaction was monitored by TLC. Furthermore, the intermediate 3 was reacted with various sulfonyl chlorides (R-SO_2_-Cl) in DCM to obtain piperazine derivatives such as S1, S3, S4, and S7 (Figure 1).

**Figure 2:**
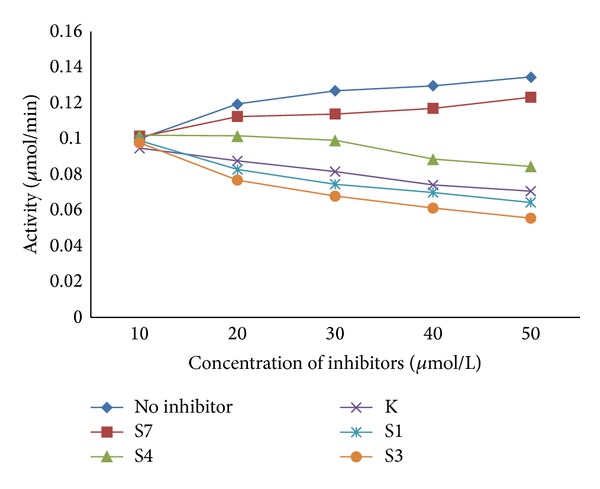
Effect of piperazine derivatives on AChE activity by Ellman's method.

The data obtained from biological evaluation done by Ellman's method, the piperazine derivatives exhibited AChE inhibitory activity in a concentration dependent manner. Among all the derivatives S3 showed significant inhibition (Figure 2). 

Based on the results from Ellman's method, docking studies were carried out to see the detailed interactions of ligand with the enzyme. The best way to fit ligand molecules (derivatives of piperazine), into AChE structure, by using Autodock4.2 resulted in docking files that contained detailed records of docking. The run with the lowest binding energy conformation in all clusters was considered as the most favorable docking pose. Binding energies that are reported are representing the sum of the total intermolecular energy, total internal energy, and torsional free energy minus the energy of the unbound system. The obtained docked files were read and converted to PDB format. Then, the docked PDB files were employed by using software LigPlot+ v.1.4, (graphical front-end to the LIGPLOT and DIMPLOT programs) for further analysis. The software automatically generates schematic diagrams of protein-ligand interactions for a given ligand in a PDB file and the number of hydrogen and hydrophobic interaction was predicted.

Docking analysis was carried out for both PAS and catalytic site, and it was observed that most of the derivatives are binding with hydrogen bonds and hydrophobic interactions to the biologically involved residues of both sites. AChE active center, which consists of the catalytic triad (Ser203, Glu334, and His447) in mammals [47], proved to be effected with the competitive inhibitors while others can influence steady state parameters by associating with an allosteric PAS site remote from the active center [48–50].

**Figure 3:**
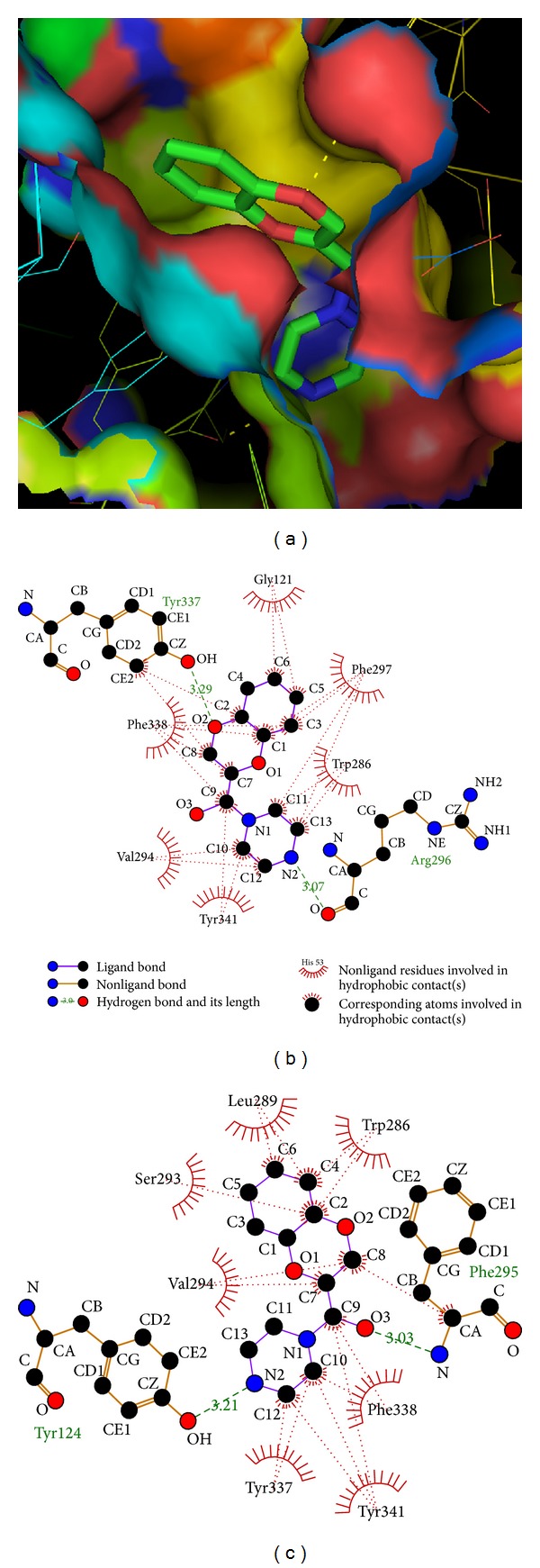
(a) Catalytic site docking of 1-(1,4-benzodioxane-2-carbonyl) piperazine (K)[Pymol view]. (b) Catalytic site docking of 1-(1,4-benzodioxane-2-carbonyl) piperazine (K). (c) Peripheral anionic site docking of 1-(1,4-benzodioxane-2-carbonyl) piperazine (K).

On catalytic site docking of the ligand K (Figure 3(a)), 2 hydrogen bonds were formed (Table 4), one between the O2 atom of the ligand and hydroxyl group of the TYR 337 residue (biologically active residue of the PAS site) at a bond length of 3.29 Å. Another hydrogen bond is formed between the N2 atom of the ligand and carboxylic group of the ARG 296 residue of the enzyme at a bond length of 3.07 Å (Figure 3(b), Table 1). Similarly, the same ligand was docked at PAS site, formed 2 hydrogen bonds (Table 5), one between the alpha amide nitrogen atom of the acyl binding pocket residue PHE 295 and O3 atom of the ligand at a bond length of 3.03 Å. Another is formed between the hydroxyl group of active PAS site residue TYR 124 and N2 atom of the ligand at a bond length of 3.21 Å (Figure 3(c), Table 1). On sum up, ligand K bonds only to the PAS site like the known allosteric inhibitors decidium (DI), propidium (PI), ethidium, and gallamine (GAL) [36–38, 48] salicylanilide N-Alkylcarbamates [23]. 

**Figure 4:**
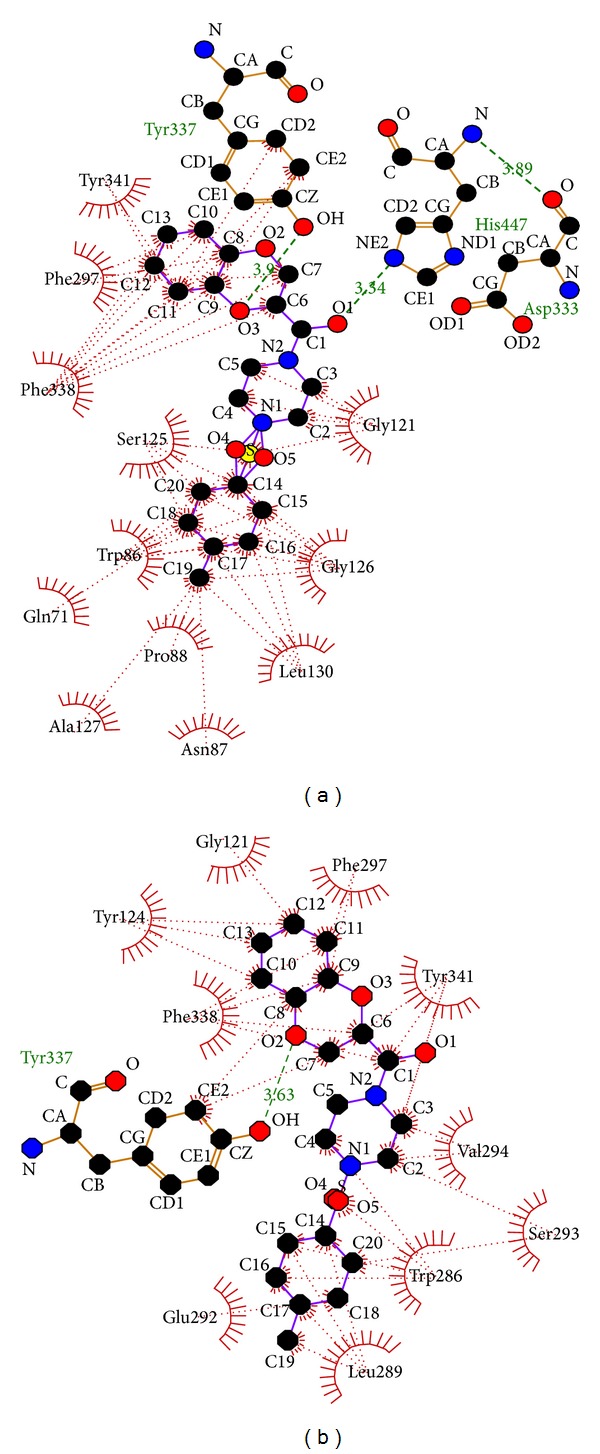
(a) Catalytic site docking of 4-(4-methyl)-benzenesulfonyl-1-(1,4-benzodioxane-2-carbonyl) piperazine (S1). (b) Peripheral anionic site docking of 4-(4-methyl)-benzenesulfonyl-1-(1,4-benzodioxane-2-carbonyl) piperazine (S1).

Likewise, the ligand S1 formed 2 hydrogen bonds on catalytic site docking (Table 4), one between the O1 atom of the ligand and N*ε*2 of the HIS 447 residue; main residue of catalytic triad is at a bond length of 3.34 Å. Another hydrogen bond is formed between the O3 atom of the ligand and hydroxyl group of the TYR 337 residue, active residue of PAS site at a bond length of 3.9 Å (Figure 4(a), Table 1). Similarly the ligand S1 was docked at PAS site which formed only one hydrogen bond (Table 5) between hydroxyl group of the TYR 337 residue and O2 atom of the ligand at a bond length of 3.63 Å (Figure 4(b), Table 1).

**Figure 5:**
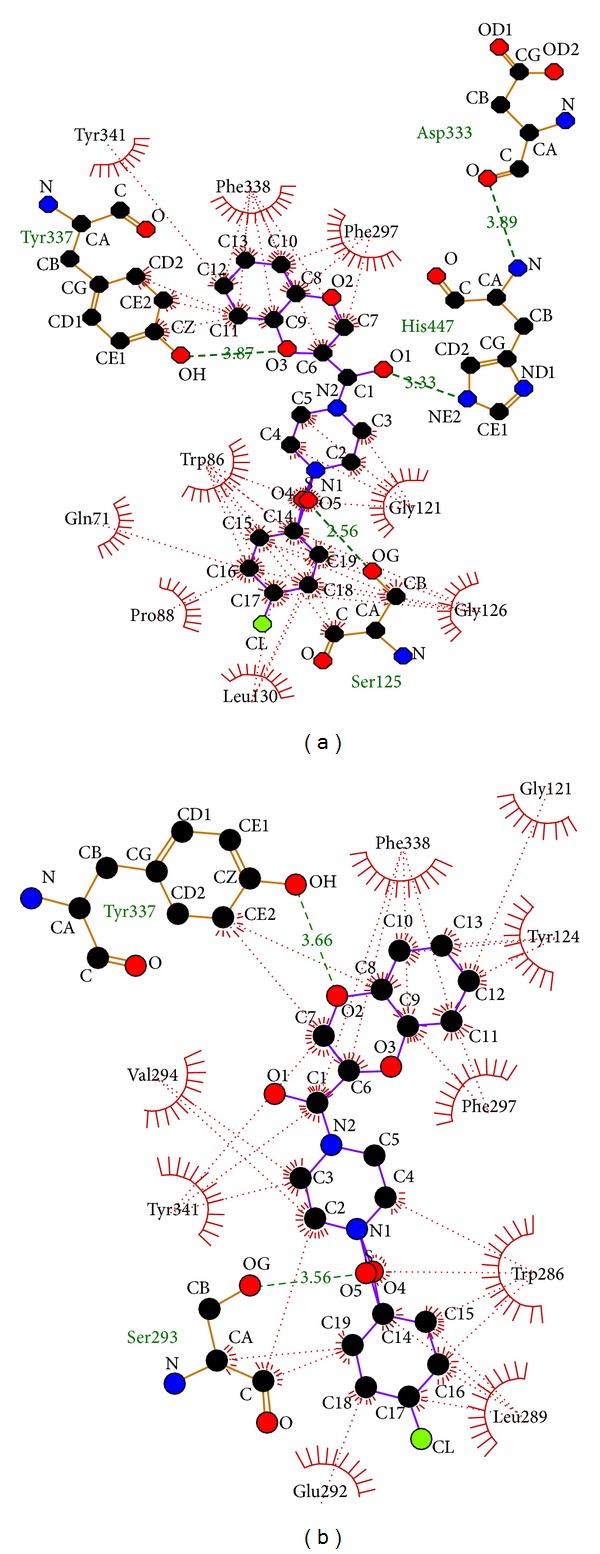
(a) Catalytic site docking of 4-(4-chloro)-benzenesulfonyl-1-(1,4-benzodioxane-2-carbonyl) piperazine (S3). (b) Peripheral anionic site docking of 4-(4-chloro)-benzenesulfonyl-1-(1,4-benzodioxane-2-carbonyl) piperazine (S3).

Correspondingly the ligand S3 formed 3 hydrogen bonds on catalytic site docking (Table 4), one between the O1 atom of the ligand and N*ε*2 of the HIS 447 residue, main residue of catalytic triad at a bond length of 3.33 Å. Another hydrogen bond is formed between the O3 atom of the ligand and hydroxyl group of the TYR 337 residue at a bond length of 3.87 Å. One more hydrogen bond is formed between the O5 atom of the ligand and hydroxyl group of the SER 125 residue; active residue of PAS site is at a bond length of 2.56 Å (Figure 5(a), Table 1). 

Equally, the ligand S3 was docked at PAS site which formed 2 hydrogen bonds (Table 5), one between the O2 atom of the ligand and hydroxyl group of the TYR 337 residue at a bond length of 3.66 Å. Whereas the other hydrogen bond is formed between the O5 atom of the ligand and hydroxyl group of the SER 293 residue at a bond length of 3.56 Å (Figure 5(b), Table 1). Totting up, the ligand S1 and S3 bind to both the residues of PAS and catalytic site (Tyr337 and His447) (Table 1) which showed a similar kind of binding pattern to Donepezil [39, 51] and act as dual-site binding inhibitors. The other reported dual site inhibitors are 7-methoxytacrine-adamantylamine heterodimers [24] and 2,3-dihydro-1H-cyclopenta[b] quinoline derivatives [52].

**Figure 6:**
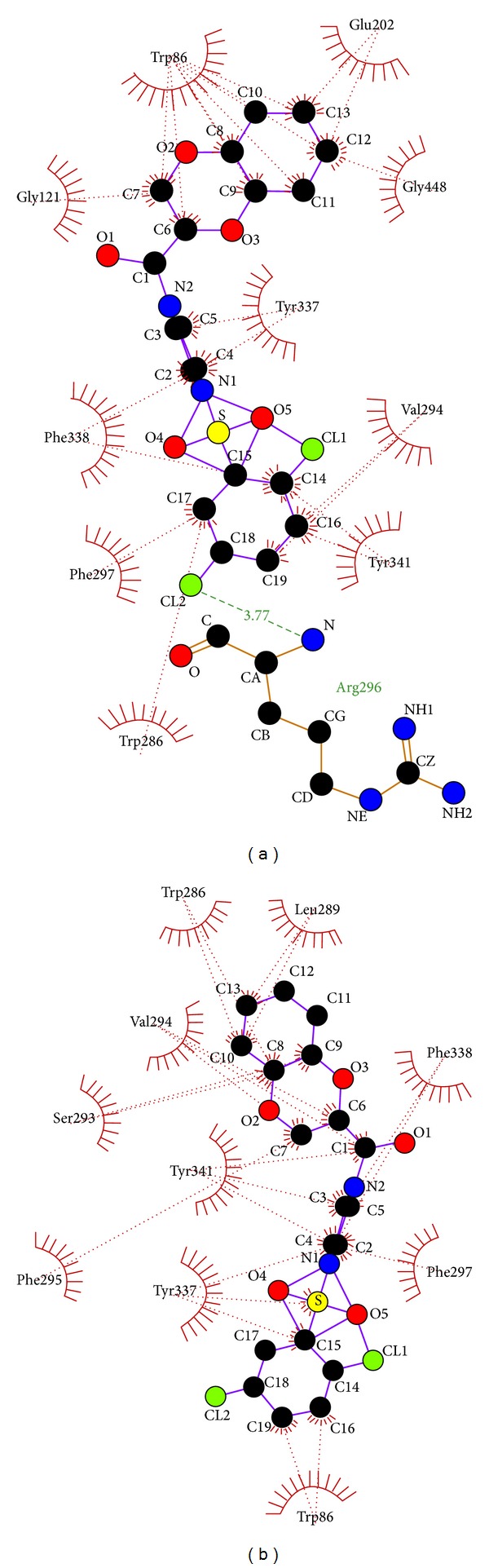
(a) Catalytic site docking of 4-(2,5-dichloro)-benzenesulfonyl-1-(1,4-benzodioxane-2-carbonyl) piperazine (S7). (b) Peripheral anionic site docking of 4-(2,5-dichloro)-benzenesulfonyl-1-(1,4-benzodioxane-2-carbonyl) piperazine (S7).

**Figure 7:**
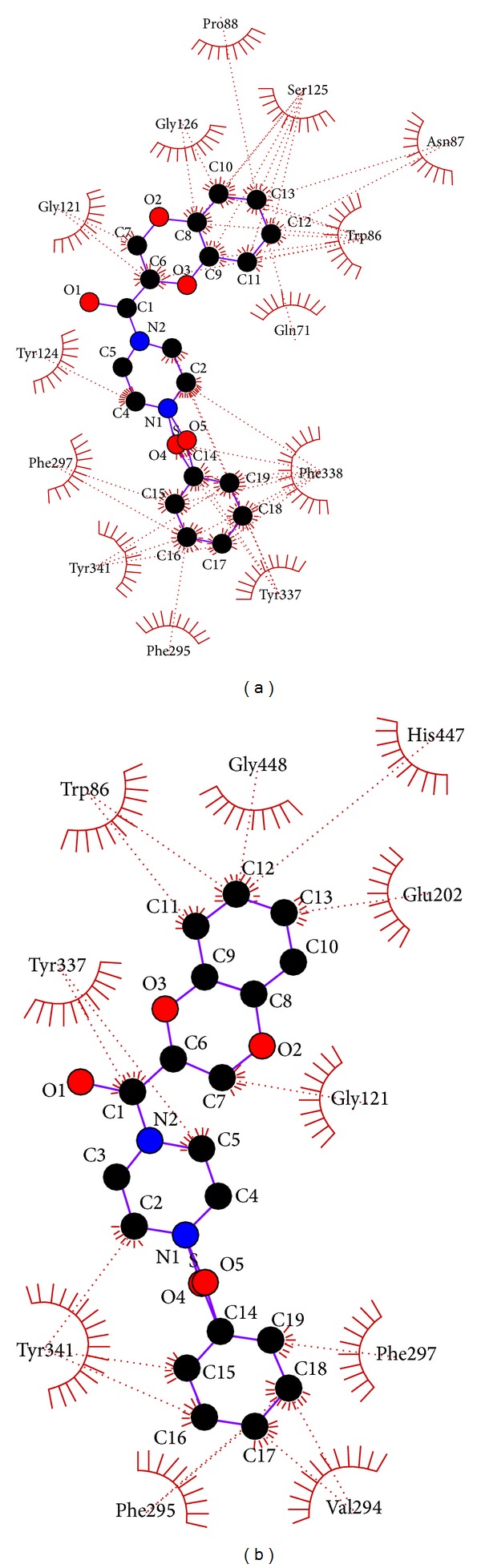
(a) Catalytic site docking of 4-benzenesulfonyl-1-(1,4-benzodioxane-2-carbonyl) piperazine (S4). (b) Peripheral anionic site docking of 4-benzenesulfonyl-1-(1,4-benzodioxane-2-carbonyl) piperazine (S4).

In the same way, the S7 forms only one hydrogen bond on catalytic site docking (Table 4) that is between alpha amide nitrogen atom of ARG 296 and second chlorine atom of ligand at a bond length of 3.77 Å (Figure 6(a), Table 1) but there is no hydrogen bond formation on PAS site docking (Figure 6(b), Tables 1 and 5). Furthermore, ligand S4 did not form hydrogen bond with any of the residues on docking at both PAS and catalytic site, even though it forms major hydrophobic interactions with the biologically active residues (Figures 7(a) and 7(b), Table 1). All these 5 ligands also formed number of hydrophobic interaction with majority of the biologically active residues (Tables 4 and 5). 

Docking results also generated the inhibition constants (*K*_*i*_) and free energy of binding (Δ*G*) values (Table 2). Among all the derivatives, S7 showed the lowest free energy of binding and the highest number of hydrophobic interactions. The Δ*G* and *K*_*i*_ values of the derivatives were comparable to the values of the FDA approved drugs tacrine (−6.95 kcal/mol, 8.03 *μ*M), rivastigmine (−5.61 kcal/mol, 77.72 *μ*M), and galantamine (−7.86 kcal/mol, 1.73 *μ*M) [53].

Furthermore, the derivatives also have drug likeness by obeying the Lipinski's rule of five (Table 3); rule with five properties to predict the chemical compound with a certain pharmacological or biological activity that would make it a likely orally active drug in humans. The derivatives were also checked for ability to permeate through intestine and blood brain barrier (BBB) by calculating the permeability factor and topological polar surface area (TPSA) [46, 54]. 

Among all the derivatives, ligand K (50.8 Å^2^) was predicted to be highly permeable as the calculated TPSA value (in Å squared) was below 70 Å^2^ (Table 3) as its suggested that molecules greater than 140 Å^2^ will have poor permeability through intestine and BBB. Till recent discoveries, the vast majority of CNS drugs have a TPSA value below 70 Å^2^ [54]. Consequently, the ligand K value with acceptable TPSA value is comparable to the FDA approved drugs (TPSA value in brackets) tacrine (38.9 Å^2^), rivastigmine (32.8 Å^2^), galantamine (41.9 Å^2^) and donepezil (38.8 Å^2^). 

## 4. Conclusion

This paper reports the biological and computational analysis of the synthesized novel piperazine derivatives as potent AChE inhibitors. The ligands S1 and S3 showed the qualities of being a dual site inhibitor. Furthermore, all the derivatives illustrated drug likeness representing the oral activeness in humans. The ligand K is predicted to be permeable through intestine and blood brain barrier on the basis of TPSA value. And, thus, the derivatives have the therapeutic potential for the treatment of Alzheimer's disease.

## Figures and Tables

**Table 1:** Details of molecular interactions in the binding site and PAS sites of docked complexes.

Ligands	PAS site				Catalytic site			
	Residue	No. of H bonds	No. of hydrophobic interactions	Site of H bond	Residues	No. of H bonds	No. of hydrophobic interactions	Site of H bond
K	PHE 295 TYR 124	2	7	*α*N*-O3^+^ OH*-N2^+^	TYR 337 ARG 296	2	6	OH*-O2^+^ C=O*-N2^+^
S1	TYR 337	1	10	OH*-O2^+^	HIS 447 TYR 337	2	12	N*ε*2*-O1^+^ OH*-O3^+^
S3	SER 293 TYR 337	2	9	OG*-O5^+^ OH*-O2^+^	HIS 447 TYR 337 SER 125	3	9	N*ε*2*-O1^+^ OH*-O3^+^ OG*-O5^+^
S4	—	0	10	—	—	0	13	—
S7	—	0	10	—	ARG 296	1	10	*α*N*-CL2^+^

*Atom from amino acid residues, ^+^atom from ligand.

**Table 2:** Comparisons of free energy of binding and inhibition constants of the derivatives on both PAS and catalytic site.

Drug (ligand) molecule	PAS site			Catalytic site		
	Estimated free energy of binding (Δ*G*), kcal/mol	Estimated inhibition constant (*K*_*i*_) *µ*M	Total intermolecular energy, kcal/mol	Estimated free energy of binding (Δ*G*), kcal/mol	Estimated inhibition constant, (*K*_*i*_) *µ*M	Total intermolecular energy, kcal/mol
1-(1,4-Benzodioxane-2-carbonyl)piperazine-K	−8.15	1.06	−8.45	−7.33	4.27	−7.62
4-(4-Methyl)-benzenesulfonyl-1-(1,4-benzodioxane-2-carbonyl) piperazine-S1	−9.90	0.05578	−10.79	−4.33	672.74	−5.22
4-Benzenesulfonyl-1-(1,4-benzodioxane-2-carbonyl) piperazine-S4	−9.79	0.06643	−10.69	−6.65	13.45	−7.54
4-(2,5-Dichloro)-benzenesulfonyl-1-(1,4-benzodioxane-2-carbonyl) piperazine-S7	−11.42	0.00426	−12.02	−11.42	0.00424	−12.02
4-(4-Chloro)-benzenesulfonyl-1-(1,4-benzodioxane-2-carbonyl) piperazine-S3	−9.97	0.04881	−10.87	−4.05	1080	−4.94

**Table 3:** Lipinski's rule of five and *topological polar surface area (TPSA), permeability factor values of derivatives.

Ligand	Num_H acceptors	Num_H donors	MiLog *P*	Number of rotatable bonds	Molecular Wt In daltons	TPSA* in Å^2^
K	5	1	0.104	1	248.28	50.804
S1	7	0	2.056	3	402.46	76.157
S4	7	0	1.608	3	388.44	76.157
S7	7	0	2.892	3	457.33	76.157
S3	7	0	2.286	3	422.88	76.157

**Table 4:** The residues from each HuAChE active site subsites (Wiesner et al., [27]) and its interaction with derivatives at catalytic site docking.

Biologically active sites of HuAChE	Residues	Catalytic site docking				
		K	S1	S3	S4	S7
Catalytic triad	Ser 203	—	—	—	—	—
	Glu 334	—	—	—	—	—
	His 447	—	H-bond	H-bond	—	—

Oxyanion hole	Gly 121	Hydrophobic	Hydrophobic	Hydrophobic	Hydrophobic	—
	Gly 122	—	—	—	—	—
	Ala 204	—	—	—	—	—

Anionic subsite	Trp 86	—	Hydrophobic	Hydrophobic	—	Hydrophobic
	Tyr 133	—	—	—	—	—
	Gly 202	—	—	—	—	Hydrophobic
	Gly 448	—	—	—	—	Hydrophobic
	Ile 451	—	—	—	—	—

Acyl binding pocket	Trp 236	—	—	—	—	—
	Phe 295	—	—	Hydrophobic	Hydrophobic	—
	Phe 297	Hydrophobic	Hydrophobic	Hydrophobic	Hydrophobic	Hydrophobic
	Phe 338	Hydrophobic	Hydrophobic	—	Hydrophobic	Hydrophobic

Peripheral anionic site	Asp 74	—	—	—	—	—
	Tyr 124	—	—	—	Hydrophobic	—
	Ser 125	—	Hydrophobic	H-bond	Hydrophobic	—
	Trp 286	Hydrophobic	—	—	—	—
	Tyr 337	H-bond	H-bond	H-bond	Hydrophobic	Hydrophobic
	Tyr 341	Hydrophobic	Hydrophobic	—	Hydrophobic	Hydrophobic

**Table 5:** The residues from each HuAChE active site subsites (Wiesner et al., [27]) and its interaction with derivatives at pas site docking.

Biologically active sites of HuAChE	Residues	PAS site docking				
		K	S1	S3	S4	S7
Catalytic triad	Ser 203	—	—	—	—	—
	Glu 334	—	—	—	—	—
	His 447	—	—	—	Hydrophobic	—

Oxyanion hole	Gly 121	—	Hydrophobic	Hydrophobic	Hydrophobic	—
	Gly 122	—	—	—	—	—
	Ala 204	—	—	—	—	—

Anionic subsite	Trp 86	—	—	—	Hydrophobic	Hydrophobic
	Tyr 133	—	—	—	—	—
	Gly 202	—	—	—	Hydrophobic	—
	Gly 448	—	—	—	Hydrophobic	—
	Ile 451	—	—	—	—	—

Acyl binding pocket	Trp 236	—	—	—	—	—
	Phe 295	H-bond	—	—	Hydrophobic	Hydrophobic
	Phe 297	—	Hydrophobic	Hydrophobic	Hydrophobic	Hydrophobic
	Phe 338	—	Hydrophobic	Hydrophobic	—	Hydrophobic

Peripheral anionic site	Asp 74	—	—	—	—	—
	Tyr 124	H-bond	—	Hydrophobic	—	—
	Ser 125	—	—	—	—	—
	Trp 286	Hydrophobic	Hydrophobic	hydrophobic	—	Hydrophobic
	Tyr 337	Hydrophobic	H-bond	H-bond	Hydrophobic	Hydrophobic
	Tyr 341	Hydrophobic	Hydrophobic	Hydrophobic	Hydrophobic	Hydrophobic

## Abbreviations

AChE: Acetylcholinesterase
TPSA: Topological polar surface area
FDA: Food and Drug Administration
AD: Alzheimer's disease
huAChE: Human acetylcholinesterase
PAS: Peripheral anionic site
PDB: Protein data bank
DMF: Dimethylsulfamide
DCM: Dichloromethane.

## References

[1] Francis PT, Palmer AM, Snape M, Wilcock GK (1999). The cholinergic hypothesis of Alzheimer’s disease: a review of progress. *Journal of Neurology Neurosurgery and Psychiatry*.

[2] Desgranges B, Baron J, de la Sayette V (1998). The neural substrates of memory systems impairment in Alzheimer’s disease. A PET study of resting brain glucose utilization. *Brain*.

[3] Förstl H, Hentschel F, Sattel H (1995). Age associated memory impairment and early Alzheimer’s diseases. *Drug Research*.

[4] McGuffey EC (1997). Alzheimer’s disease: an overview for the pharmacist. *The Journal of the American Medical Association*.

[5] Contestabile A (2011). The history of the cholinergic hypothesis. *Behavioural Brain Research*.

[6] Weinstock M (1997). Possible role of the cholinergic system and disease models. *Journal of Neural Transmission, Supplement*.

[7] Becker R, Giacobini E, Elble R, McIlhany M, Sherman K (1988). Potential pharmacotherapy of Alzheimer disease. A comparison of various forms of physostigmine administration. *Acta Neurologica Scandinavica*.

[8] Parnetti L, Senin U, Mecocci P (1997). Cognitive enhancement therapy for Alzheimer’s disease. The way forward. *Drugs*.

[9] Brinton RD, Yamazaki RS (1998). Advances and challenges in the prevention and treatment of Alzheimer’s disease. *Pharmaceutical Research*.

[10] Berkheij M (2005). Synthesis of 2-substituted piperazines via direct *α*-lithiation. *Tetrahedron Letters*.

[11] Upadhayaya RS, Sinha N, Jain S, Kishore N, Chandra R, Arora SK (2004). Optically active antifungal azoles: synthesis and antifungal activity of (2R,3S)-2-(2,4-difluorophenyl)-3-(5-{2-[4-aryl-piperazin-1-yl]-ethyl} -tetrazol-2-yl/1-yl)-1-[1,2,4]-triazol-1-yl-butan-2-ol. *Bioorganic and Medicinal Chemistry*.

[12] Chaudhary P, Kumar R, Verma AK (2006). Synthesis and antimicrobial activity of N-alkyl and N-aryl piperazine derivatives. *Bioorganic and Medicinal Chemistry*.

[13] Mallesha L, Mohana KN (2011). Synthesis, antimicrobial and antioxidant activities of 1-(1, 4-benzodioxane-2-carbonyl)piperazine derivatives. *European Journal of Chemistry*.

[14] Rossen K, Weissman SA, Sager J (1995). Asymmetric hydrogenation of tetrahydropyrazines: synthesis of (*S*)-piperazine-2-tert-butylcarboxamide, an intermediate in the preparation of the HIV protease inhibitor indinavir. *Tetrahedron Letters*.

[15] Torisu K, Kobayashi K, Iwahashi M (2004). Discovery of a new class of potent, selective, and orally active prostaglandin D2 receptor antagonists. *Bioorganic and Medicinal Chemistry*.

[16] Bolchi C, Pallavicini M, Fumagalli L (2005). Highly efficient resolutions of 1,4-benzodioxane-2-carboxylic acid with para substituted 1-phenylethylamines. *Tetrahedron Asymmetry*.

[17] Marchini N, Bombieri G, Artali R, Bolchi C, Pallavicini M, Valoti E (2005). Influence of (*S*)-1-phenylethylamine para substitution on the resolution of (±)-1,4-benzodioxane-2-carboxylic acid: a crystallographic, theoretical and morphologic approach. *Tetrahedron Asymmetry*.

[18] Fang QK, Grover P, Han Z (2001). Practical chemical and enzymatic technologies for (*S*)-1,4-benzodioxan-2-carboxypiperizine intermediate in the synthesis of (*S*)-doxazosin mesylate. *Tetrahedron Asymmetry*.

[19] Sepčić K, Marcel V, Klaebe A, Turk T, Šuput D, Fournier D (1998). Inhibition of acetylcholinesterase by an alkylpyridinium polymer from the marine sponge, *Reniera sarai*. *Biochimica et Biophysica Acta*.

[20] Wang H, Chou C, Liao J, Chen C (2001). Dehydroevodiamine attenuates *β*-amyloid peptide-induced amnesia in mice. *European Journal of Pharmacology*.

[21] Guevara-Salazar JA, Espinoza-Fonseca M, Beltrán HI, Correa-Basurto J, Zavala DQ, Trujillo-Ferrara JG (2007). The electronic influence on the active site-directed inhibition of acetylcholinesterase by N-aryl-substituted succinimides. *Journal of the Mexican Chemical Society*.

[22] Wang YH, Wan QL, Gu CD, Luo HR, Long CL (2012). Synthesis and biological evaluation of lycorine derivatives as dual inhibitors of human acetylcholinesterase and butyrylcholinesterase. *Chemistry Central Journal*.

[23] Imramovsky A, Stepankova S, Vanco J (2012). Acetylcholinesterase-inhibiting activity of salicylanilide N-alkylcarbamates and their molecular docking. *Molecules*.

[24] Spilovska K, Korabecny J, Kral J (2013). 7-Methoxytacrine-adamantylamine heterodimers as cholinesterase inhibitors in Alzheimer's disease treatment—synthesis, biological evaluation and molecular modeling studies. *Molecules*.

[25] Grisaru D, Sternfeld M, Eldor A, Glick D, Soreq H (1999). Structural roles of acetylcholinesterase variants in biology and pathology. *European Journal of Biochemistry*.

[26] Sussman JL, Harel M, Frolow F (1991). Atomic structure of acetylcholinesterase from *Torpedo californica*: a prototypic acetylcholine-binding protein. *Science*.

[27] Wiesner J, Kriz Z, Kuca K, Jun D, Koca J (2007). Acetylcholinesterases—the structural similarities and differences. *Journal of Enzyme Inhibition and Medicinal Chemistry*.

[28] Hosea NA, Radić Z, Tsigelny I, Berman HA, Quinn DM, Taylor P (1996). Aspartate 74 as a primary determinant in acetylcholinesterase governing specificity to cationic organophosphonates. *Biochemistry*.

[29] Radic Z, Reiner E, Taylor P (1991). Role of the peripheral anionic site on acetylcholinesterase: inhibition by substrates and coumarin derivatives. *Molecular Pharmacology*.

[30] Johnson G, Moore SW (1999). The adhesion function on acetylcholinesterase is located at the peripheral anionic site. *Biochemical and Biophysical Research Communications*.

[31] Muñoz FJ, Aldunate R, Inestrosa NC (1999). Peripheral binding site is involved in the neurotrophic activity of acetylcholinesterase. *NeuroReport*.

[32] Inestrosa NC, Alvarez A, Pérez CA (1996). Acetylcholinesterase accelerates assembly of amyloid-*β*-peptides into Alzheimer’s fibrils: possible role of the peripheral site of the enzyme. *Neuron*.

[33] Inestrosa NC, Alarcón R (1998). Molecular interactions of acetylcholinesterase with senile plaques. *Journal of Physiology Paris*.

[34] Bourne Y, Taylor P, Bougis PE, Marchot P (1999). Crystal structure of mouse acetylcholinesterase: a peripheral site-occluding loop in a tetrameric assembly. *The Journal of Biological Chemistry*.

[35] Soreq H, Seidman S (2001). Acetylcholinesterase—new roles for an old actor. *Nature Reviews Neuroscience*.

[36] Taylor P, Lappi S (1975). Interaction of fluorescence probes with acetylcholinesterase. The site and specificity of propidium binding. *Biochemistry*.

[37] Berman HA, Decker MM (1986). Kinetic, equilibrium, and spectroscopic studies on dealkylation (“aging”) of alkyl organophosphonyl acetylcholinesterase. Electrostatic control of enzyme topography. *The Journal of Biological Chemistry*.

[38] Berman HA, Decker MM, Nowak MW (1987). Site selectivity of fluorescent bisquaternary phenanthridinium ligands for acetylcholinesterase. *Molecular Pharmacology*.

[39] Kryger G, Silman I, Sussman JL (1999). Structure of acetylcholinesterase complexed with E2020 (Aricept): implications for the design of new anti-Alzheimer drugs. *Structure*.

[40] Greenblatt HM, Guillou C, Guénard D (2004). The complex of a bivalent derivative of galanthamine with torpedo acetylcholinesterase displays drastic deformation of the active-site gorge: implications for structure-based drug design. *Journal of the American Chemical Society*.

[41] Alonso D, Dorronsoro I, Rubio L (2005). Donepezil-tacrine hybrid related derivatives as new dual binding site inhibitors of AChE. *Bioorganic and Medicinal Chemistry*.

[42] Ellman GL, Courtney KD, Andres V, Featherstone RM (1961). A new and rapid colorimetric determination of acetylcholinesterase activity. *Biochemical Pharmacology*.

[43] Morris GM, Goodsell DS, Halliday RS (1998). Automated docking using a Lamarckian genetic algorithm and an empirical binding free energy function. *Journal of Computational Chemistry*.

[44] Lipinski CA (2000). Drug-like properties and the causes of poor solubility and poor permeability. *Journal of Pharmacological and Toxicological Methods*.

[45] Lipinski CA, Lombardo F, Dominy BW, Feeney PJ (1997). Experimental and computational approaches to estimate solubility and permeability in drug discovery and development settings. *Advanced Drug Delivery Reviews*.

[46] Ertl P, Rohde B, Selzer P (2000). Fast calculation of molecular polar surface area as a sum of fragment-based contributions and its application to the prediction of drug transport properties. *Journal of Medicinal Chemistry*.

[47] Rachinsky TL, Camp S, Li Y, Ekstrom TJ, Newton M, Taylor P (1990). Molecular cloning of mouse acetylcholinesterase: tissue distribution of alternatively spliced mRNA species. *Neuron*.

[48] Changeux JP (1966). Responses of acetylcholinesterase from *Torpedo marmorata* to salts and curarizing drugs. *Molecular Pharmacology*.

[49] Roufogalis BD, Quist EE (1972). Relative binding sites of pharmacologically active ligands on bovine erythrocyte acetylcholinesterase. *Molecular Pharmacology*.

[50] Robaire B, Kato G (1974). Effects of Mg^2+^ and Ca^2+^ on soluble and membrane bound acetylcholinesterase from *Electrophorus electricus*. *Biochemical Pharmacology*.

[51] Melnikova I (2007). Therapies for Alzheimer’s disease. *Nature Reviews Drug Discovery*.

[52] Szymanski P, Laznickova A, Laznicek M, Bajda M, Malawska B, Markowicz M (2012). 2, 3-dihydro-1H-cyclopenta[b]quinoline derivatives as acetylcholinesterase inhibitors-synthesis, radiolabeling and biodistribution. *International Journal of Molecular Sciences*.

[53] Sharma J, Ramanathan K, Sethumadhavan R (2011). Identification of potential inhibitors against acetylcholinesterase associated with Alzheimer's diseases: a molecular docking approach. *Journal of Computational Methods in Molecular Design*.

[54] Veber DF, Johnson SR, Cheng H, Smith BR, Ward KW, Kopple KD (2002). Molecular properties that influence the oral bioavailability of drug candidates. *Journal of Medicinal Chemistry*.

